# Clinical research on the efficacy and safety of thread-embedding acupuncture for treatment of herniated intervertebral disc of the lumbar spine: a protocol for a multicenter, randomized, patient–assessor blinded, controlled, parallel, clinical trial

**DOI:** 10.1186/s13063-018-2864-4

**Published:** 2018-09-10

**Authors:** Bonhyuk Goo, Dek-Woo Ryoo, Eun-Jung Kim, Dongwoo Nam, Hyun-Jong Lee, Jae-Soo Kim, Yeon-Cheol Park, Yong-Hyeon Baek, Byung-Kwan Seo

**Affiliations:** 1 Department of Clinical Korean Medicine, Graduate School, Kyung Hee University, 26, Kyungheedae-ro, Dongdaemun-gu, Seoul, 02447 Republic of Korea; 2 Department of Acupuncture & Moxibustion Medicine, College of Oriental Medicine, Dongguk University, 123, Dongdae-ro, Gyeongju-si, Gyeongsangbuk-do 38066 Republic of Korea; 3 Department of Acupuncture & Moxibustion Medicine, College of Korean Medicine, Kyung Hee University, 26, Kyungheedae-ro, Dongdaemun-gu, Seoul, 02447 Republic of Korea; 4 Department of Acupuncture & Moxibustion medicine, College of Korean medicine, Daegu Haany University, 136, Sincheondong-ro, Suseong-gu, Daegu, 42158 Republic of Korea; 5 Department of Acupuncture & Moxibustion, Kyung Hee University Hospital at Gangdong, 892, Dongnam-ro, Gangdong-gu, Seoul, 05278 Republic of Korea

**Keywords:** Thread-embedding acupuncture, Lumbar herniated intervertebral disc, Lumbar disc herniation, Lower back pain, Radiculopathy

## Abstract

**Background:**

A lumbar herniated intervertebral disc (LHIVD) is a common problem that usually causes lower back pain and neurological symptoms that manifest as radiating pain. Several studies have reported that thread-embedding acupuncture (TEA) is effective in the treatment of LHIVD. However, these studies were of low quality and there is therefore little clinical evidence for the effectiveness of TEA in this regard. The aim of the present study is to establish the clinical evidence regarding the efficacy and safety of TEA in the treatment of pain, function, and quality of life in patients with LHIVD. The study uses a rigorously designed, full-scale, randomized clinical trial (RCT) protocol.

**Method/design:**

This is a multicenter, randomized, patient–assessor blinded, sham-controlled trial with two parallel arms. Seventy patients with LHIVD who have lower back pain more severe than 40 mm on the 100-mm visual analogue scale (VAS), and who are aged 19–70 years, will be recruited and randomly allocated to a TEA group and sham TEA (STEA) group. Both groups will receive treatment on 23 predefined acupoints once a week for eight weeks; needles with the thread removed will be used in the STEA group, while normal TEA will be employed in the TEA group. Changes in the 100-mm VAS for lower back pain after eight weeks will be assessed as the primary outcome. Furthermore, the 100-mm VAS for radiating pain, Oswestry Disability Index, Roland–Morris disability questionnaire, EuroQol 5 Dimensions 5 Levels, and global perceived effect will be measured and analyzed as secondary outcomes. All outcomes will be assessed at baseline and at 4, 8, 12, and 16 weeks after screening.

**Discussion:**

The results of this trial will help to establish the clinical evidence regarding TEA in the treatment of patients with LHIVD.

**Trial registration:**

ClinicalTrials.gov, NCT03236753. Registered on August 2, 2017.

Clinical Research Information Service of the Republic of Korea, KCT0002439. Registered on August 1, 2017.

**Electronic supplementary material:**

The online version of this article (10.1186/s13063-018-2864-4) contains supplementary material, which is available to authorized users.

## Background

A lumbar herniated intervertebral disc (LHIVD) is a common problem leading to lower back pain and neurological symptoms, including radiating pain in the lower extremities [1]. After other diseases are ruled out, such as lumbar stenosis, spondylolisthesis, and fracture, approximately 85% of patients suffering from lower back pain and radiculopathy are found to have an LHIVD [2]; the annual incidence is five per 1000 adults [3].

Depending on the severity of the neurological deficit, either surgical or conservative treatments may be considered [4]. Conservative methods, such as non-steroidal anti-inflammatory drugs, epidural injections, physical therapy, and alternative treatments are appropriate for most patients who do not have severe neurological symptoms [5, 6]. In addition, complementary and alternative medicine may be an option for patients with LHIVD, and the use of herbal medicine, cupping, manipulation, and various types of acupuncture has increased [7, 8].

In particular, thread-embedding acupuncture (TEA) involves embedding absorbable foreign substances, such as catgut and polydioxanone sutures, into acupoints using needles. The technique utilizes long-term stimulation, unlike traditional acupuncture [9]. Embedding a foreign substance also adds chemical stimulation to the mechanical stimulation of traditional acupuncture [10]. TEA has been used to treat a variety of diseases, including obesity [11], allergic rhinitis [12], facial palsy [13], and LHIVD. Although several recent randomized clinical trials (RCTs) have reported that TEA has a more favorable therapeutic effect on LHIVD than other types of acupuncture or other treatments, the evidence remains limited because these trials used poor assessment methods and had a high risk of bias [14].

To establish the clinical evidence regarding the efficacy and safety of TEA in the treatment of LHIVD, we will compare TEA with a sham control in a rigorously designed, full-scale RCT protocol that includes data on pain, function, and quality of life (QoL).

## Methods/design

### Trial design

**Fig. 1:**
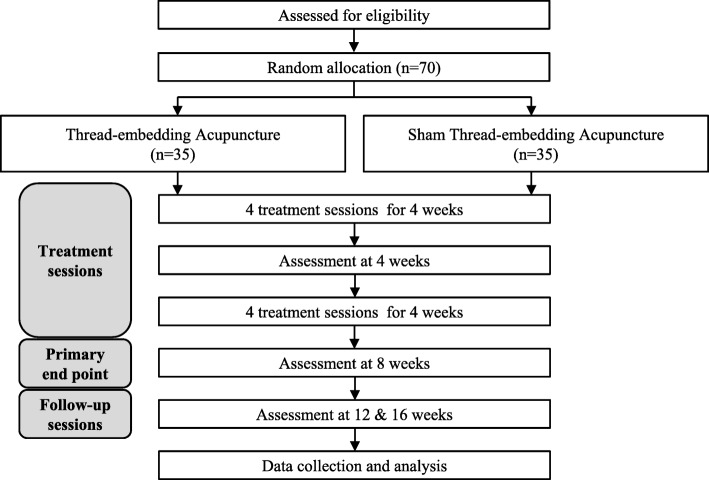
Study flowchart

This clinical study is a multicenter, randomized, patient–assessor blinded, sham-controlled trial with two parallel arms (1:1 ratio). The trial will take place at the Kyung Hee University Hospital, Gangdong (KHUHGD), Kyung Hee University Medical Center (KHUMC), Dongguk University Bundang Oriental Hospital (DUBOH), and Daegu Korean Medicine Hospital of Daegu Haany University (DKMHDHU). The efficacy and safety of TEA in patients with LHIVD will be evaluated by comparison with sham TEA (STEA) (Fig. 1).

The protocol of this study has been approved by the Institutional Review Boards (IRBs) of the institutions (KHUHGD: KHNMCOH 2016-09-006, KHUMC: 161216-HR-006, DUBOH: 2016–0012, DKMHDHU: DHUMC-D16015-PRO-02). The trial has also been registered at clinicaltrials.gov, which is a website of the United States National Institutes of Health (registration number: NCT03236753), as well as with the Clinical Research Information Service of the Republic of Korea (registration number: KCT0002439). All research procedures comply with Korean Good Clinical Practice (KGCP) and the Declaration of Helsinki. The methodology was established in accordance with the Consolidated Standards of Reporting Trials (CONSORT) [15], as well as with the revised Standards for Reporting Interventions in Clinical Trials of Acupuncture (STRICTA) [16]. Standard Protocol Items: Recommendations for Interventional Trials (SPIRIT) checklist is attached as Additional file 1 [17].

### Participants

The inclusion criteria for this study are as follows: (1) men or women aged 19–70 years; (2) radiating pain combined with abnormality on the lumbar spine that is more severe than bulging, as shown by computed tomography or magnetic resonance imaging [18, 19]; (3) ≥ 40 mm pain intensity on the 100-mm pain visual analogue scale (VAS) for lower back pain; and (4) agreement to participate and signing of the informed consent form after a detailed explanation of the clinical trial.

Participants who have the following characteristics will be excluded: (1) congenital abnormalities or surgical history in the lumbar region; (2) red flag signs that may indicate *cauda equina* syndrome, such as bladder and bowel dysfunction or saddle anesthesia; (3) tumor, fracture, or infection in the lumbar region; (4) injection in the lumbar region within one week of the trial start; (5) psychiatric disorder currently being treated, such as depression or schizophrenia; (6) inappropriate conditions for TEA due to skin disease or hemostatic disorder (prothrombin time with an international normalized ratio > 2.0 or treatment using an anticoagulant); (7) other diseases that could affect or interfere with therapeutic outcomes, including severe gastrointestinal disease, cardiovascular disease, hypertension, diabetes, renal disease, liver disease, or thyroid disorder; (8) contraindications for acetaminophen, including concurrent disease, hypersensitivity reaction, or other medication; (9) pregnancy or other inappropriate condition for TEA; (10) heavy drinking (> 3 glasses per day), which can cause hepatotoxicity when combined with acetaminophen [20].

### Procedure

**Fig. 2:**
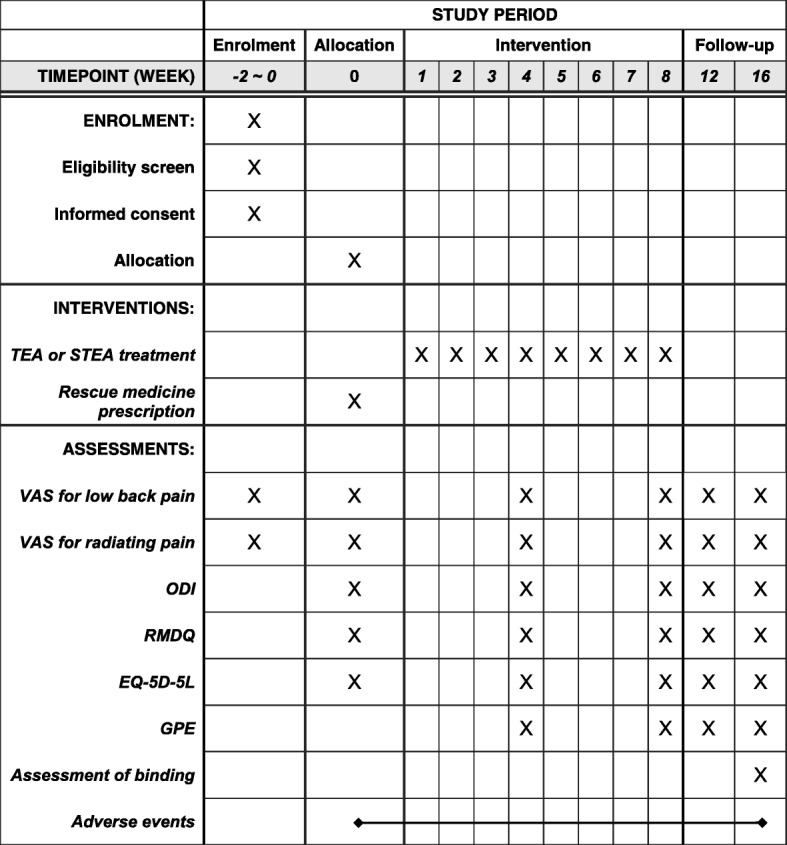
Standard Protocol Items: Recommendations for Interventional Trials (SPIRIT) figure. TEA thread-embedding acupuncture, STEA sham thread-embedding acupuncture, VAS visual analogue scale, ODI Oswestry Disability Index, RMDQ Roland-Morris disability questionnaire, EQ-5D-5 L EuroQol 5 Dimensions 5 Levels, GPE global perceived effect

Seventy participants with LHIVD will be recruited at four sites, each of which will recruit an appointed number of patients (KHUHGD: 18, KHUMC: 18, DUBOH: 18, DKMHDHU: 16). All participants who can read and write in Korean will be informed that they may voluntarily participate and that they can withdraw their consent at any stage. They will also be given essential information regarding the study protocol, including purpose, selection of participants, interventions by random allocation, schedule, expected benefits and risks, alternative treatment options, and confidentiality. Those who agree and sign the informed consent form will be screened through the assessment of demographics, medical history, present illness, vital sign, pregnancy test, and laboratory test. If they meet the eligibility criteria, the participants will be randomly allocated to the TEA group or STEA group. After random allocation, 10 visit sessions for treatment and assessment will be conducted according to an appointed 16-week schedule (Fig. 2).

### Interventions

In both groups, the intervention will be administered once a week for eight weeks using either a 29-gauge, 40-mm or a 29-gauge, 60-mm TEA or STEA needle (Hyundae Meditech, Wonju, South Korea). The treatment will be applied on 23 predefined acupoints according to STRICTA (Table 1). After sterilization of the skin surface, participants will be administered TEA in a prone position. In the STEA group, thread-removed TEA will be used instead of normal TEA. The thread will be removed aseptically and secretly to prevent infection and ensure patient-blinding, respectively.

**Table 1:** Details of TEA treatment using the STRICTA 2010 checklist

Item	Detail
1. Acupuncture rationale	1a. Style of acupuncture: Thread-embedding acupuncture (TEA)
	1b. Reasoning for treatment provided (based on historical context, literature sources, and/or consensus methods, with references where appropriate): By the consensus of a group of clinical experts, based on a previous study [21–24]
	1c. Extent to which treatment was varied: Using bilateral EX-B2 of the herniated intervertebral disc its upper and lower levels
2. Details of needling	2a. Number of needle insertions per subject per session: 23 acupoints
	2b and 2c. Names of points used and depth of insertion, based on a specified unit of measurement, or on a particular tissue level: Bilateral EX-B2 of the level of the herniated intervertebral disc: perpendicular insertion (4 cm) Bilateral EX-B2 of the upper level of the herniated intervertebral disc: perpendicular insertion (4 cm) Bilateral EX-B2 of the lower level of the herniated intervertebral disc: perpendicular insertion (4 cm) Bilateral BL24: transverse insertion towards the L1 level along the erector muscle of the spine (6 cm) Bilateral BL25: transverse insertion towards the iliac crest (4 cm) Bilateral BL26: transverse insertion towards the L1 level along the erector muscle of spine (6 cm) Bilateral BL26: oblique insertion towards the iliolumbar ligament (6 cm) Bilateral EX-B7: oblique insertion towards the gluteus medius muscle (6 cm) Bilateral BL28: oblique insertion towards the sacroiliac ligament (6 cm) Bilateral GB30: perpendicular insertion (6 cm) Unilateral (symptomatic side) GB34, BL57, and ST36: oblique insertion towards the foot (4 cm)
	2d. Response sought: None
	2e. Needle stimulation: No additional stimulation
	2 f. Needle retention time: None
	2 g. Needle type: TEA (29-gauge × 40 mm, or 29-gauge × 60 mm)
3. Treatment regimen	3a. Number of treatment sessions: Eight
	3b. Frequency and duration of treatment sessions: Once a week for 8 weeks
4. Other components of treatment	4a. Details of other interventions administered to the acupuncture group: Rescue medication, lifestyle, education
	4b. Setting and context of treatment, including instructions to practitioners, and information and explanations to patients: Minimized conversation between practitioner and participant
5. Practitioner background	5. Description of participating acupuncturists: Specialists from the acupuncture and moxibustion department or residents supervised by a specialist with at least 3 years of clinical experience
6. Control or comparator interventions	6a. Rationale for the control or comparator in the context of the research question, with sources that justify this choice: Thread-removed TEA will be used as a comparator. In this way, the study focuses specifically on the existence of thread.
	6b. Precise description of the control or comparator. If sham acupuncture or any other type of acupuncture-like control is used (provide details as for Items 1 to 3 above): All conditions other than the use of thread-removed TEA will be the same as those in the treatment group.

*STRICTA* Standards for Reporting Interventions in Clinical Trials of Acupuncture, *TEA* thread-embedding acupuncture

The selection of acupoints and details of the procedure have been modified from similar studies [21–24] by a committee of Korean medicine doctors (KMDs) experienced in LHIVD and TEA. All therapeutic procedures will be performed by KMDs who have completed or have been taking a specialist acupuncture and moxibustion course for at least three years. To ensure homogeneity of intervention among the participating centers, all practitioners will undertake a training course together in advance.

### Rescue medication and concurrent treatment

Rescue medication will be provided to participants during the study period. If they feel severe pain (> 60 mm on the 100-mm VAS), they will be permitted to take up to 2600 mg/day (four 650-mg tablets) and 6500 mg/week (10 tablets) of acetaminophen (Suspen ER C.T.; 650 mg/tablet, Hanmi Pharm. Co., Ltd., Seoul, Republic of Korea). A clinical research coordinator (CRC) will educate the participants in keeping a medication diary; they will also check the returned medications. To precisely assess efficacy, rescue medicine will be prohibited within 24 h of a visit.

Other than rescue medication, all treatment that may affect the result will be restricted, including injections, surgical interventions, acupuncture, physical therapy, or drugs for pain control. Medications that the participants have been taking before the trial, or those that they take for other diseases or adverse events (AE), will be allowed at the discretion of the investigators depending on whether they affect the symptoms of LHIVD. Changes to concurrent treatments will be recorded at each visit.

### Outcomes

The primary outcome will be change in the intensity of lower back pain. The secondary outcomes will be intensity of radiating pain, functions of daily life, QoL, and global perceived effect (GPE). Additionally, all AEs will be recorded at each visit and assessment of blinding will be conducted at the end of the study. All assessments will be performed by independent researchers who are not involved in the scheduled intervention (Table 2).

**Table 2:** Schedule for assessment

Outcome measures	Screening	Baseline	Treatment (weeks 1–8)		Follow-up (weeks 9–16)	
			Week 4	Week 8	Week 12	Week 16
VAS for lower back pain	◎	◎	◎	◎	◎	◎
VAS for radiating pain		◎	◎	◎	◎	◎
ODI		◎	◎	◎	◎	◎
RMDQ		◎	◎	◎	◎	◎
EQ-5D-5 L		◎	◎	◎	◎	◎
GPE			◎	◎	◎	◎
Assessment of binding						◎
Adverse events^a^		◎	◎	◎	◎	◎

^a^Adverse event will be monitored throughout the study period

*VAS* visual analogue scale, *ODI* Oswestry Disability Index, *RMDQ* Roland-Morris disability questionnaire, *EQ-5D-5 L* EuroQol 5 Dimensions 5 Levels, *GPE* global perceived effect

### Primary outcome measure

The intensity of lower back pain will be evaluated using the 100-mm VAS [25]. At pre-trial screening, at weeks 1 (baseline), 4, and 8 (primary endpoint), and at weeks 12 and 16 (follow-up sessions), participants will be asked to record their pain intensity within the past week on a 100-mm linear scale (0, absence of pain; 100, worst pain imaginable). Change from baseline after eight sessions of treatment will be compared between the groups as the primary outcome.

### Secondary outcome measures

#### 100-mm VAS for radiating pain

The intensity of radiating pain of the lower extremities will also be assessed using a 100-mm VAS at pre-trial screening and at weeks 1, 4, 8, 12, and 16.

#### Oswestry Disability Index

Inability to function in daily life due to lower back pain will be assessed using the ODI [26] at weeks 1, 4, 8, 12, and 16. The ODI questionnaire consists of 10 sections: pain; personal care; lifting; walking; sitting; standing; sleeping; sex life; social life; and traveling. Each question is scored from 0 to 5; the total score is calculated as a percentage disability.

#### Roland-Morris disability questionnaire

Dysfunction in daily life will be assessed using the Roland-Morris disability questionnaire (RMDQ) at weeks 1, 4, 8, 12, and 16. The RMDQ contains 24 sentences that people have used to describe themselves when they have lower back pain. Participants check the sentences that they think best describe their life on that day; the score is the total number of sentences checked (from 0 to 24) [27].

#### EuroQol 5 Dimensions 5 Levels

The patients’ QoL and general health status will be assessed using the EuroQol 5 Dimensions 5 Levels (EQ-5D-5 L) at weeks 1, 4, 8, 12, and 16. The EQ-5D-5 L essentially consists of the EQ-5D descriptive system and EQ VAS. The EQ-5D descriptive system comprises five dimensions: mobility; self-care; usual activities; pain/discomfort; and anxiety/depression. Each dimension is rated from 1 to 5 (1, no problems; 2, slight problems; 3, moderate problems; 4, severe problems; 5, extreme problems). The EQ VAS scale can be used to assess a patient’s current health status. It is a 20-cm scale numbered from 0 to 100 (0, the worst health they can imagine; 100, the best health they can imagine) [28].

#### Global perceived effect

The subjective effects of the treatments will be assessed using the GPE at weeks 4, 8, 12, and 16. The participants will score their perceived change using a 7-point scale (1, worst ever; 2, much worse; 3, worse; 4, not improved and not worse; 5, improved; 6, much improved; 7, best ever) [29].

#### Assessment of blinding

At the end of the study, the researchers will assess the appropriateness of the patient-blinding method by asking the participants which group they think they belong to. The answer can take three forms as follows: “TEA;” “STEA;” or “do not know.” The blinding index (BI) will be calculated using James’ method based on the answer. The BI increases from 0 to 1 with the success of patient-blinding (0, total lack of blinding; 0.5, completely random; 1, complete blinding) [30, 31].

### Sample size

To calculate the adequate sample size, the expert group set the difference of primary outcome measure between the two groups to 10 mm with the standard deviation of 13.66 on the basis of a previous similar study [32]. With 1:1 ratio, 80% power, and a 0.05 significance level for the independent t-test, we calculated the sample size using the formula below (α = 0.05, β = 0.2, σ = 0.3.66 and *d* = 10):

$$ \mathrm{n}=2{\sigma}^2{\left({\mathrm{Z}}_{\alpha /2}+{\mathrm{Z}}_{\beta}\right)}^2/{d}^2 $$

Considering a 10% dropout rate and 95% compliance to the calculated value, we determined that adequate sample size in each group is 35 participants. The 70 participants will be recruited separately at the four research sites; each site will recruit the following numbers of participants: KHUHGD, 18; KHUMC, 18; DUBOH, 18; DKMHDHU, 16.

### Randomization and allocation concealment

At each institution, the 70 participants will be randomly allocated to the TEA group or STEA group according to a randomization procedure with a 1:1 ratio. The randomization sequence will be generated by an independent statistician using the randomization program of the statistical analysis system. Next, sealed, opaque envelopes containing random code will be sent to each institution. The CRC will open the envelope and allocate participants to their groups. To conceal the allocation until the end of the study, information about the allocation will be recorded in a separate log and provided only to those researchers who are carrying out the intervention.

### Blinding

To achieve patient–assessor blinding, only the CRC will handle the allocation information. The CRC will provide restricted information in accordance with the researchers’ role in the study. We will explain to the participants only that they will be treated using one of the two interventions: TEA or STEA. Each participant will be treated at a different time to prevent an exchange of information. The participants in each group will receive treatment at the same sites and they will be placed in a prone position so that they cannot see the procedure. Practitioners will minimize their conversation with the participants. The researchers conducting the assessment will be blinded to the allocation, and they will only ask about what is essential to complete the case report form (CRF). An independent statistician will analyze the research data and will not be given information about the allocation.

### Statistical methods

The data will be corrected using the “last observation carried forward” method and then analyzed using the “intention-to-treat” principle. The independent t-test and Chi-square test will be used to compare differences in general characteristics between the groups. As a primary outcome, changes in the 100-mm VAS for lower back pain from baseline to the end of the eighth treatment will be compared between the groups using the independent t-test. To compare the outcomes at each session with the baseline values, an analysis of covariance will be used. Trends over time and time-by-treatment interactions will be analyzed using a repeated measures analysis of variance. All statistical analyses will be performed using PASW statistics 18 for Windows, and the statistical significance level will be considered to be 0.05 (two-sided), with 95% confidence intervals.

### Data collection and management

The following data will be collected and cross-checked by two independent researchers using the CRF: characteristics of participants; outcomes; and AEs. All data and documents obtained during the study period will be confidentially managed according to the standard operation protocol (SOP) of the IRBs. No private data unrelated to the study will be collected and personally identifiable information will be discarded after a certain period, in accordance with the SOP. The researchers will be given training in protecting the privacy of participants.

### Safety

At the pre-trial screening, the following tests will be performed: complete blood count; liver and renal function tests; blood coagulation test; urinalysis; and pregnancy test (if necessary). Information about expected AEs, as well as a contact number, will be given to the participants along with the informed consent form before the pre-trial screening. If AEs do occur, the principal investigator will evaluate the severity of the incident, as well as its relation to the interventions. They will then provide proper examination and treatment in accordance with the compensation rules. Particularly, in the case of serious AEs, the participants will be removed from the study. The progress of all AEs will be recorded in the CRF and handled in accordance with the regulation of KGCP.

### Quality control

To maintain the quality of the trial, the study procedure and documents will be periodically monitored by the Korean Medicine Clinical Trial Center (K-CTC).

## Discussion

This RCT will be conducted in a rigorous, sham-controlled setting to investigate the therapeutic effects of TEA. TEA has been reported to have beneficial effects on musculoskeletal diseases such as ankylosing spondylitis [33], chronic pelvic pain [34], and cervical spondylosis [35], and a review study on the use of TEA to treat LHIVD reported that 17 RCTs had positive results. However, these RCTs had a high risk of bias, especially in terms of blinding, because they used an active control [14]. TEA differs from traditional acupuncture because it involves both chemical and mechanical stimulation using embedded substances. Therefore, sham controls using thread-removed TEA are appropriate in attempting to discover whether the treatment has any unique effects.

To blind patients, the thread will be removed from the needles in a sterile manner with the help of an assistant. In this way, the patients will not see the thread removal. This method was found by Yoo et al. to be safe [36]. However, it will also be necessary to verify whether the blinding has actually worked. Furthermore, to blind the assessors, pre-assigned independent assessors will refrain from conversations with the practitioners that are unrelated to the evaluation. All data will be organized and analyzed by an independent statistician in accordance with the study protocol.

The TEA procedure and the selection of acupoints were decided by the consensus of a committee of clinical experts, with reference to former RCTs [21–24] and specified whole details, as prescribed in the STRICTA form. To standardize the procedures, details of the acupuncture, including size, acupoints, direction, sessions, frequency, and duration are specified in the protocol and will be communicated to all practitioners, who are acupuncture specialists, in a preliminary meeting for practice. All researchers participating in this study also completed the KGCP training course from the certificated institution of the Korean Ministry of Food and Drug Safety.

The results of this study will yield clinical evidence comparing TEA to needle-only acupuncture by providing data about subjective changes in lower back pain, radiating pain, global function, and QoL in patients with LHIVD over time. These findings will help clinicians to understand TEA and utilize the treatment as a therapeutic option for patients with LHIVD.

### Trial status

This study began on 11 August 2017, after IRB approval. The estimated study completion date is 30 June 2018.

## Additional file

Additional file 1: SPIRIT 2013 Checklist: Recommended items to address in a clinical trial protocol and related documents*. (DOC 120 kb)

## Abbreviations

AE: Adverse event
BI: Blinding index
CONSORT: Consolidated Standards of Reporting Trials
CRC: Clinical research coordinator
CRF: Case Report Form
DKMHDHU: Daegu Korean Medicine Hospital of Daegu Haany University
DUBOH: Dongguk University Bundang Oriental Hospital
EQ-5D-5 L: EuroQol 5 Dimensions 5 Levels
GPE: Global perceived effect
IRB: Institutional Review Board
K-CTC: Korean Medicine Clinical Trial Center
KGCP: Korean Good Clinical Practice
KHUHGD: Kyung Hee University Hospital at Gangdong
KHUMC: Kyung Hee University Medical Center
KMD: Korean medicine doctor
LHIVD: Lumbar herniated intervertebral disc
ODI: Oswestry Disability Index
QoL: Quality of life
RCT: Randomized clinical trial
RMDQ: Roland-Morris disability questionnaire
SOP: Standard operation protocol
SPIRIT: Standard Protocol Items: Recommendations for Interventional Trials
STEA: Sham thread-embedding acupuncture
STRICTA: Standards for Reporting Interventions in Clinical Trials of Acupuncture
TEA: Thread-embedding acupuncture
VAS: Visual analogue scale
